# Reconstructing the recent West Nile virus lineage 2 epidemic in Europe and Italy using discrete and continuous phylogeography

**DOI:** 10.1371/journal.pone.0179679

**Published:** 2017-07-05

**Authors:** Gianguglielmo Zehender, Carla Veo, Erika Ebranati, Valentina Carta, Francesca Rovida, Elena Percivalle, Ana Moreno, Davide Lelli, Mattia Calzolari, Antonio Lavazza, Chiara Chiapponi, Laura Baioni, Gioia Capelli, Silvia Ravagnan, Graziana Da Rold, Enrico Lavezzo, Giorgio Palù, Fausto Baldanti, Luisa Barzon, Massimo Galli

**Affiliations:** 1 Department of Biomedical and Clinical Sciences "L.Sacco", University of Milan, Milano, Italy; 2 CRC-Coordinated Research Center “EpiSoMI”, University of Milan, Milano, Italy; 3 Molecular Virology Unit, Microbiology and Virology Department, Fondazione IRCCS Policlinico San Matteo, Pavia, Italy; 4 Experimental Zooprophylactic Institute of Lombardy and Emilia-Romagna (IZSLER), Brescia, Italy; 5 Experimental Zooprophylactic Institute of Lombardy and Emilia-Romagna (IZSLER), Reggio Emilia, Italy; 6 Experimental Zooprophylactic Institute of Lombardy and Emilia-Romagna (IZSLER), Parma, Italy; 7 Experimental Zooprophylactic Institute of Venice, Legnaro, Padua, Italy; 8 Department of Molecular Medicine, University of Padova, Padova, Italy; National and Kapodistrian University of Athens, GREECE

## Abstract

West Nile virus lineage 2 (WNV-2) was mainly confined to sub-Saharan Africa until the early 2000s, when it was identified for the first time in Central Europe causing outbreaks of human and animal infection. The aim of this study was to reconstruct the origin and dispersion of WNV-2 in Central Europe and Italy on a phylodynamic and phylogeographical basis. To this aim, discrete and continuous space phylogeographical models were applied to a total of 33 newly characterised full-length viral genomes obtained from mosquitoes, birds and humans in Northern Italy in the years 2013–2015 aligned with 64 complete sequences isolated mainly in Europe. The European isolates segregated into two highly significant clades: a small one including three sequences and a large clade including the majority of isolates obtained in Central Europe since 2004. Discrete phylogeographical analysis showed that the most probable location of the root of the largest European clade was in Hungary a mean 12.78 years ago. The European clade bifurcated into two highly supported subclades: one including most of the Central/East European isolates and the other encompassing all of the isolates obtained in Greece. The continuous space phylogeographical analysis of the Italian clade showed that WNV-2 entered Italy in about 2008, probably by crossing the Adriatic sea and reaching a central area of the Po Valley. The epidemic then spread simultaneously eastward, to reach the region of the Po delta in 2013, and westward to the border area between Lombardy and Piedmont in 2014; later, the western strain changed direction southward, and reached the central area of the Po valley once again in 2015. Over a period of about seven years, the virus spread all over an area of northern Italy by following the Po river and its main tributaries.

## Introduction

West Nile virus (WNV) is a neurotropic mosquito-borne virus belonging to the Flavivirus genus and Japanese encephalitis virus serogroup [1]. Its genome is represented by a single-stranded positive-sense RNA molecule coding for a single polyprotein encompassing a total of three structural proteins corresponding to the viral core, membrane and envelope, and seven non-structural proteins. WNV is maintained by an enzootic cycle involving birds (particularly birds of the *Passeriformes* order and migratory birds) and ornithophilic mosquitoes such as species of the *Culex* genus. Humans, horses and other mammals are dead-end hosts that may be incidentally involved in the enzootic cycle. Most human infections are asymptomatic, whereas fever may occur in 20% of cases and neuroinvasive disease in less than 1% [1].

WNV was first isolated in a febrile woman in Uganda in 1937 [2], and sporadic cases and small outbreaks were documented in Africa, the Middle East and Europe during the 1950s-1960s [3]. In 1996, a major outbreak in Romania was characterized by a high (10%) fatality rate and more than 350 people with neurological symptoms [4]; since then, there have been a number of equine or human outbreaks in various parts of the Mediterranean area and central Europe [5, 6]. In 1999, the virus was introduced into New York City, where it caused a dramatic outbreak that spread throughout the entire Western hemisphere in subsequent years **{CDC, 1999 #41;** [7].

Phylogenetic studies of WNV have revealed the existence of at least eight evolutionary lineages, of which lineages 1 and 2 represent the most important human pathogens [6].

Lineage 1 is widespread in all continents, whereas lineage 2 was mainly confined to sub-Saharan Africa until the early 2000s, when the virus emerged in eastern Europe: two different strains of lineage 2 were respectively detected for the first time in Hungary and southern Russia in 2004, and led to outbreaks among wild birds with some cases of human infection [8, 9].

The first human cases of infection due to WNV-2 in Europe were reported in Hungary in 2008 [10].

In the following years, WNV-2 has been regularly identified every year as the cause of local outbreaks or sporadic cases of infection throughout east and central Europe [11, 12] causing in particular, the explosive 2010 outbreak in northern Greece that led to 262 human cases, 197 of whome developed severe WN neurological diseases and 33 died [13] and the 300 human infections notified in Serbia in 2013 [6]. The Russian strain, which has been responsible for human outbreaks in the Volgograd and Astrakhan regions since 2007, has also spread to Europe; it has been involved in human infections in Romania since 2010 [14] and, in 2014, it was detected in mosquito pools collected in north-eastern Italy but was not associated with human infections [15]. WNV lineage 2 is currently responsible for the vast majority of human WNV infections reported in Europe.

WNV was detected in Italy for the first time in 1998, during an outbreak among horses in Tuscany [16], and then re-appeared ten years later in north-eastern Italy, where a number of cases of human or horse infections are reported every year [17]. Various strains of WNV lineage 1 (the Western Mediterranean clade) have been involved in human infection in Italy since 1998, whereas WNV lineage 2 was documented for the first time in 2011 [18–20]. However, it has since spread throughout the country and is now the most frequently identified lineage in Italy [19, 21, 22].

A “phylodynamic” approach [23] may describe the correlations between the eco/epidemiology and evolutionary processes of highly evolving viruses, thus allowing the reconstruction of the history of an infectious agent on the basis of the phylogeny of sampled sequences. In particular, phylogeographical analysis allows reconstruction on a geographical scale and may provide important information about emerging or re-emerging infectious diseases by determining when and from which reservoir it emerged, and how it spreads.

Phylogeographic methods most frequently employed are based on models considering locations as discrete traits. Recently, models based on the use of geographic coordinates (longitude and latitude) of the sampling locations, allow the reconstruction of the phylogeography in a bidimensional continuous space, without the uncertainty due to the necessity to build discrete groups.

The aim of this study was to reconstruct the origin and the dispersion routes of WNV-2 in Europe (particularly in Italy) in order to provide useful information for its surveillance. A classical discrete and a continuous phylogeographical methods have been used to describe the local spread of WNV-2 in Europe and in Italy respectively.

## Materials and methods

### Patients and datasets

The study was conducted using 33 newly characterised complete genome viral sequences obtained from mosquito (n = 7), bird (n = 21) and human samples (n = 5). The human samples were obtained from a starting population of 21 patients with WNV disease and 6 blood donors. All human samples were tested for serology (identification of IgM and IgG immunoglobulins and identification and titration of neutralizing antibodies with neutralization assays) and molecular biology (Real-time PCR) at the clinical and transfusional centres. Based on the results of these tests and clinical symptoms, patients and donors were defined “probable cases” and “confirmed cases”, referring to the clinical and diagnostic criteria established by the European Union in 2008 for the definition of probable and confirmed cases of WNV virus infection (European Centre for Disease Prevention and Control, ECDC).

The in vitro isolation of the virus was possible for 4 patients and 1 blood donor from which the sequences included in the study were obtained.

The sampling period ranged from 2013 to 2015, and sampling areas included Cremona, Brescia, Modena, Ferrara, Mantua, Reggio Emilia, Lodi, Milan, Parma, and Pavia.

Mosquito samples were sampled in georeferenced stations within the framework of the national surveillance system targeting West Nile virus. The trapped insects were kept in the original sampling bag and refrigerated to preserve the WNV from degradation. The mosquitoes were identified at species level by means of morphological keys [24], and grouped in pools of 100 units on the basis of the date of collection, location, and species. Only pools of *Culex pipiens*, a major vector of the virus, were analysed.

The bird samples were collected between May and November within the framework of wildlife population control programmes authorised by the Italian Institute for Environmental Protection and Research (ISPRA). The carcasses of the birds, mainly magpies (*Pica pica*), hooded crows (*Corvus corone cornix*) and Eurasian jays (*Garrulus glandarius*), were collected and delivered to the laboratory by rangers and hunters as target species for WNV. The wild birds positive for WNV belonged to seven species, with magpies (9 strains) and hooded crows (4 strains) being the most frequently affected European species.

Table 1 summarises the data relating to the Italian samples newly characterized in the study.

**Table 1:** Codes, host, localities and collection years of the newly characterized WNV-2 Italian sequences included in the dataset.

Year	Codes	Host	Municipality	Province	Region
2013	41iCR@13	Culex Pipiens	Soresina	Cremona	LO
2013	42iCR@13	Crow (Corvus cornix)	Rivolta d'Adda	Cremona	LO
2013	43iBS@13	Culex Pipiens	Leno	Brescia	LO
2013	171iMO@13	Magpie (Pica pica)	Castelfranco Emilia	Modena	ER
2013	172iFE@13	Upupa (Upupa epops)	Ferrara	Ferrara	ER
2013	173iFE@13	Jay (Garrulus glandarius)	Ferrara	Ferrara	ER
2013	174iFE@13	Blackbird (Turdus merula)	Ferrara	Ferrara	ER
2013	175iFE@13	Magpie (Pica pica)	Poggio Renatico	Brescia	LO
2013	176iFE@13	Assiolo (Otus scops)	Ferrara	Ferrara	ER
2013	177iMN@13	Mosquitoes (Culex pipiens)	San Giovanni del Dosso	Mantova	LO
2013	178iRE@13	Magpie (Pica pica)	Novellara	Reggio Emilia	ER
2014	179iLO@14	Mosquitoes (Culex pipiens)	Borgetto Lodigiano	Lodi	LO
2014	180iLO@14	Mosquitoes (Culex pipiens)	Borgetto Lodigiano	Lodi	LO
2014	181iMI@14	Crow (Corvus cornix)	Milano	Milano	LO
2014	182iMO@14	Magpie (Pica pica)	San Cesario sul Panaro	Modena	ER
2014	183iPR@14	Northern goshawk (Accipiter gentilis)	Noceto	Parma	ER
2015	184iFE@15	Hooded crow (Corvus corone cornix)	Bondeno	Ferrara	ER
2015	185iFE@15	Mosquitoes (Culex pipiens)	Ferrara	Ferrara	ER
2015	186iBS@15	Northern goshawk (Accipiter gentilis)	Bagnolo Mella	Brescia	LO
2015	187iPV@15	Mosquitoes (Culex pipiens)	Parona	Pavia	LO
2015	188iMO@15	Magpie (Pica pica)	San Cesario sul Panaro	Modena	ER
2015	189iMO@15	Magpie (Pica pica)	Castelfranco Emilia	Modena	ER
2015	190iAL@15	Homo Sapiens	Mortara	Pavia	LO
2015	191iLO@15	Homo Sapiens	Sant'Angelo Lodigiano	Lodi	LO
2015	192iLO@15	Homo Sapiens	Chignolo Po	Pavia	LO
2015	193iPV@15	Homo Sapiens	Redavalle	Pavia	LO
2015	194iPV@15	Homo Sapiens	Pavia	Pavia	LO
2015	199iMO@15	Magpie (Pica pica)	San Cesario sul Panaro	Modena	ER
2015	200iMO@15	Magpie (Pica pica)	Castelfranco Emilia	Modena	ER
2015	201iMO@15	Hooded crow (Corvus corone cornix)	Ravarino	Modena	ER
2015	202iMO@15	Hooded crow (Corvus corone cornix)	Ravarino	Modena	ER
2015	203iMO@15	Hooded crow (Corvus corone cornix)	Cavezzo	Modena	ER
2015	204iMO@15	Magpie (Pica pica)	Mirandola	Modena	ER

The mosquito and bird samples were analysed using a TaqMan One-Step RT-PCR for WNV as proposed by Tang *et al*. [25], and the positive samples were then tested using a real-time RT-PCR for the detection of L1 and L2 WNV genomes [26]. Whole genome sequencing of the animal isolates was performed using the Miseq sequencer (Illumina Inc. San Diego, CA, USA). Viral RNA was extracted, reverse-transcribed and amplified by means of PCR using random primers as described by [27]. After purification, amplified PCR sequencing libraries were prepared using the Nextera-XT kit (Illumina Inc., San Diego, CA, USA), suitably diluted, pooled, and sequenced. The *reads* were assembled using Lasergene v.12 (DNASTAR, Madison, USA) software and *de novo* or re-sequencing analysis with an appropriate reference virus.

The RNA from human WNV isolates was prepared by extracting it from the cell culture supernatants using the NucleoMag 96 Virus kit (Machery-Nagel, Düren, Germany), and the automatic King Fisher mL Magnetic Particle Processor (Thermo Fisher Scientific, Inc., NYSE: TMO) in accordance with the manufacturer’s protocol. The complete genome was amplified using 22 primer pairs. The amplicons were cleaned up using the QIAquick PCR Purification kit (QIAGEN, Germany) in accordance with the manufacturer's protocol, and the purified PCR products underwent nucleotide sequencing using an ABI PRISM 3130 Genetic Analyser (Applied Biosystems Inc.).

The study sequences were aligned with 64 complete genome sequences of WNV isolated in various European and African countries and retrieved from public databases (GenBank at http://www.ncbi.nlm.nih.gov/genbank/; S1 Table). The sequences were selected on the basis of the following criteria: i) they had to have been published in peer-reviewed journals; ii) their non-recombinant subtype assignment had to be certain; iii) the city/state of origin and year of sampling had to be known and clearly established in the original publication.

The sampling dates ranged from 1937 to 2015, and the sampling locations were Austria (AT, n = 4), the Central African Republic (CF, n = 1), Cyprus (CY, n = 1), the Czech Republic (CZ, n = 4), the Democratic Republic of the Congo (CD, n = 1), Greece (GR, n = 14), Hungary (HU, n = 2), Italy (IT, n = 52), Madagascar (MG, n = 1), Romania (RO, n = 1), Russia (RU, n = 1), Senegal (SN, n = 1), Serbia (SR, n = 2), South Africa (ZA, n = 8), Uganda (UG, n = 3), and Ukraine (UK, n = 1).

### Ethics statement

The local Ethics Committee consent was not required because according to a Regional Surveillance and Preparedness Plan (DGR 12591, December 27, 2012), diagnostic detection of WNV infections in the Lombardy Region was centralized at the Regional Reference Laboratory (Molecular Virology Unit, Fondazione IRCCS Policlinico San Matteo, Pavia). Informed consent was not necessary because patients with suspected WNV infections were included in a Regional diagnostic protocol. Prospective samples (serum, CSF, and urine) were collected by clinicians and handled by Molecular Virology Unit personnel; data were analyzed anonymously according to a Regional Surveillance and Preparedness Plan (DGR 12591, December 27, 2012).

### Phylogenetic analysis

The sequences were aligned using ClustalX software [28] followed by manual editing using Bioedit software, and the best fitting nucleotide substitution model was tested by means of a hierarchical likelihood ratio test (LRT) implemented in J Modeltest software [29]. The selected model was GTR with gamma-distributed rates among sites and a proportion of invariant sites.

The phylogeny of the complete WNV-2 genome was reconstructed using two different approaches: a maximum likelihood approach using a new hill-climbing algorithm implemented in PHYML [30], and a Bayesian Markov Chain Monte Carlo (MCMC) method (Mr Bayes). The reliability of the observed clades was established on the basis of internal node bootstrap values ≥70% (after 200 replicates in PHYML) or posterior probabilities with significance levels of ≥0.7.

### Likelihood mapping

The phylogenetic signal of the complete genome dataset was investigated by means of the likelihood mapping using TreePuzzle [31]. A total of 10000 random quartets (groups of four randomly chosen sequences) were evaluated and, for each quartet, the three possible unrooted trees were reconstructed using the maximum likelihood approach under the selected substitution model. The posterior probabilities of each tree were then plotted on a triangular surface: fully resolved trees fall into the corners and the unresolved quartets in the centre of the triangle. When more than 30% of the dots fall into the centre, the data are considered unreliable for phylogenetic inference.

### Root-to-tip analysis

In order to investigate the temporal structure and “clock-likeness” of the West Nile virus data set, we made a regression analysis of the root-to-tip genetic distance against sampling years using TempEst [32] and a maximum-likelihood tree without a molecular clock assumption [30].

### Bayesian phylogenetic analysis

The tree topology, substitution and coalescent models, the evolutionary rate and consequently tMRCAs were co-estimated using a Bayesian MCMC approach implemented in the Beast package version 1.8 [33] (http://beast.bio.ed.ac.uk). A strict and uncorrelated log-normal relaxed clock models were compared by Bayes factor (BF) test. A less restrictive Bayesian skyline plot (BSP) with 10 interval groups was used as the coalescent prior.

The MCMC analysis was run until convergence, and sampled every thousandth generation. Convergence was assessed by estimating the effective sampling size (ESS) after a 10% burn-in, using Tracer software version 1.6 (http://tree.bio.ed.ac.uk/software/tracer/), and ESS>200 were accepted. Uncertainty was indicated by 95% highest posterior density (95% HPD) intervals. The strength of the evidence against H_0_ was evaluated in accordance with Kass and Raftery [34] as follows: 2lnBF <2 no evidence; 2–6 weak evidence; 6–10 strong evidence, and >10 very strong evidence. A negative 2lnBF indicates evidence in favour of H_0_. Only values of ≥6 were considered significant.

The TMRCA estimates were expressed as median and 95% HPD years before the most recent sampling date, corresponding to 2015 in this study.

For the analysis of evolutionary population dynamics three simple parametric coalescent models (constant population size, and exponential and logistic growth) and the Bayesian skyline plot (BSP, a classical piece-wise constant-multiple change process) were compared [35]. The coalescent model best fitting the data was selected by means of a BF test, as also described above.

### Discrete phylogeographical analysis

The reconstruction of the ancestral discrete states was obtained by the same Bayesian framework in which the spatial diffusion of the time-scaled genealogy is modelled as a continuous-time Markov chain [36] process over discrete sampling locations. In order to find a minimal (parsimonious) set of rates explaining the diffusions in the phylogeny, a Bayesian stochastic search variable selection (BSSVS) approach was used, which allows the migration rates in the CTMC to be zero with some prior probability. Comparing the posterior to prior odds that individual rates are zero provides a Bayes factor test to identify the significant rates (BF>3) [36].

The obtained trees were summarised in a maximum clade credibility tree using the Tree Annotator program included in the Beast package and choosing the tree with the maximum product of posterior probabilities (maximum clade credibility: MCC) after a 10% burn-in. The branches of the tree were labelled with different state colours according to the most probable location. In order to visualise diffusion rates over time, it is possible to rendering the location-annotated MCC tree to a geo json data format suitable for viewing with georeferencing softwares. A new analysis tool (SPREAD3) was used [37]. The MCC tree is converted to a java script object (JSON) file. Next the JSON file is then used for rendering the visualization using a Data Driven Document (D3) library.

### Continuous phylogeographical analysis

In order to study the spread of WNV-2 in Italy in more detail, a continuous space phylogeographical analysis was made using the Italian isolates forming a single monophyletic group within the large European clade.

The coordinates of the sampling locations were available for the birds and mosquitos (traps), and were deduced on the basis of the residence of the patients at the time they developed symptoms after excluding recent travels out of Italy. A map of the area considered is represented in S1 Fig.

Italian WNV epidemics were investigated in continuous space using Beast v 1.8.0. The unknown coordinates were estimated under a strict Brownian diffusion model and compared with two relaxed random walk (RRW) models relaxing along the phylogeny the diffusion rate constancy assumption [38]. The two RRW models respectively assumed a Gamma and Cauchy distribution of diffusion rates over the phylogeny. The Bayes factor comparison between the models was made by estimating marginal likelihood using path sampling (PS) and stepping stone approaches [39].

The phylogeny was spatially projected and converted into the D3 library [37] in order to visualise dispersal over time. Uncertainty in ancestral location estimates was represented by polygons delimitating the high-probability regions.

## Results

### Phylogenetic analysis

**Fig 1:**
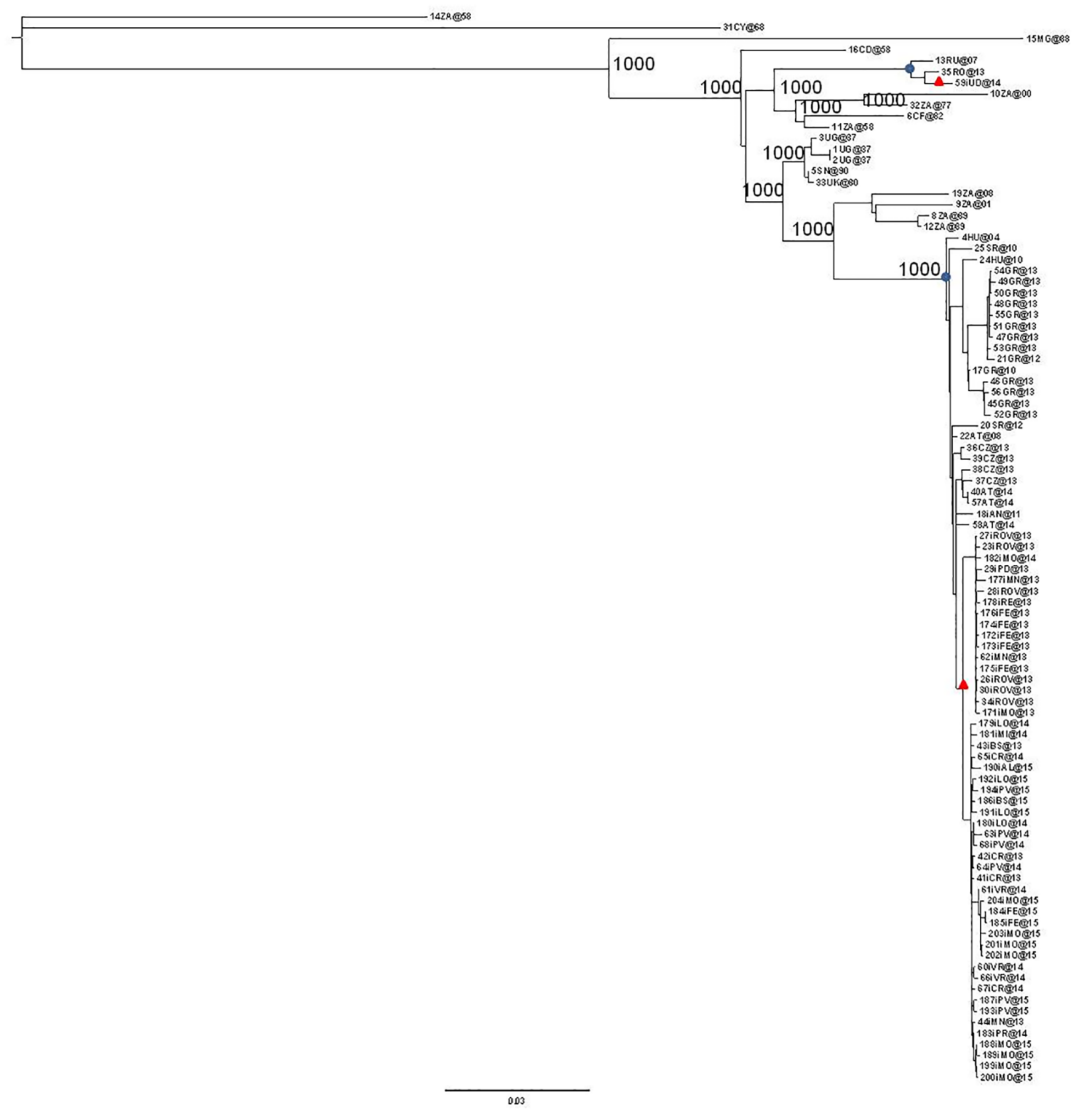
Maximum likelihood tree of the 97 West Nile virus complete genome sequences. The European clades have been highlighted (blue circles). The Italian clades are identified by red triangles. The scale bar indicates 3% of nucleotide divergence.

The entire dataset including all of the isolates was analysed using a maximum likelihood approach and Bayesian method (Fig 1 and S2 Fig), which identified a number of highly significant clades that were supported by bootstrap values of 1000 and a pp of 1 and mainly segregated geographically.

As shown in Fig 1, the majority of the European isolates belonged to a large monophyletic group supported by high posterior probability (bootstrap = 1000, pp = 1) that included most of the strains that have been circulating in Europe since 2004; only three strains (one from Russia, one from Romania, and one from Italy) segregated independently in a separate small clade (bootstrap = 1000, pp = 1).

All the newly characterised Italian isolates grouped in a highly significant sub-clade of the major European clade (bootstrap = 998, pp = 1).

### Likelihood mapping and root-to-tip regression analysis

In order to investigate the presence of phylogenetic noise, the complete genome data set was investigated by means of likelihood mapping. The fact that 5.1% of the dots fell in the central area and 93.7% at the corners of the triangles indicates that the alignment contained sufficient phylogenetic information (S3 Fig).

Root-to-tip regression analysis of the genetic distances of WNV-2 against sampling time, which was made using TempEst software, produced a correlation coefficient of 0.81 and a coefficient of determination (R^2^) of 0.66, thus suggesting a significant relationship between genetic divergence and time.

### Bayesian discrete phylogeographical analysis of the major European clade

Comparison of the strict and relaxed molecular clocks, and different coalescent models showed that the model best fitting the data was a coalescent prior BSP (2 lnBF GMRF Bayesian Skyride *vs* BSP = -564.36; 2lnBF constant *vs* BSP = -64.94; 2lnBF exponential growth *vs* BSP = -57.28; 2lnBF logistic growth *vs* BSP = -94.76) under a log-normal relaxed clock (2lnBF strict *vs* relaxed clock = -65.04).

Under these conditions, the estimated substitution rate of the entire viral genome was 5.18 x 10^−4^ (lower 95% HPD = 3.82 x 10^−4^ and upper 95% HPD = 6.61x 10^−4^).

**Fig 2:**
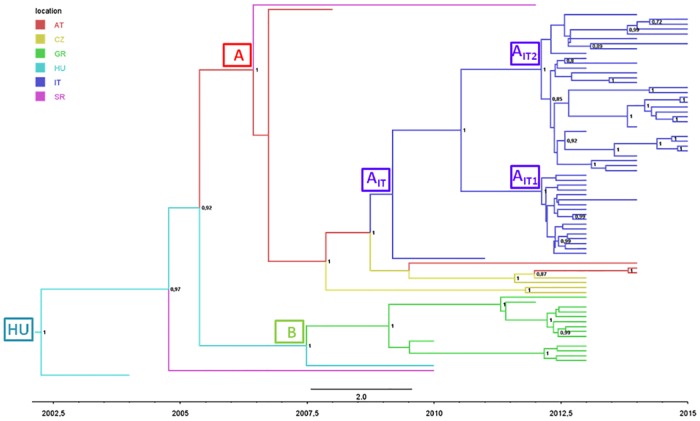
Phylogeographic analysis of 77 European WNV-2 isolates forming a single monophyletic group. The branches of the maximum clade credibility (MCC) tree are coloured on the basis of the most probable location of the descendent nodes (AT = Austria; CZ = Czech Republic; GR = Greece; HU = Hungary; IT = Italy; SR = Serbia). The numbers on the internal nodes indicate significant posterior probabilities (pp >0.7), and the scale at the bottom of the tree represents calendar years. The main geographical clades are highlighted.

Fig 2 shows the phylogeography of the largest European clade and indicates that the most probable tree-root location was Hungary (stpp = 0.80 *vs* stpp = 0.09 for Serbia, the second most probable location).

The mean root tMRCA was 12.78 YA (95%HPD: 11.00–15.06), corresponding to the year 2002. The European monophyletic group showed several highly supported clades, one of which (clade A) included isolates from several central/eastern European countries (Austria, the Czech Republic, Serbia, Italy). The second highly significant clade included all of the isolates obtained in Greece and a single Hungarian isolate (clade B). The most probable ancestral location of clade A was Austria (stpp = 0.52), and that of clade B was Hungary (stpp = 0.66). The mean tMRCA of both subclades was 2007.

Table 2 shows the estimated locations and tMRCAs of the main sub-clades.

**Table 2:** Estimated times of the most recent common ancestors (tMRCAs) of the main clades and credibility intervals (95%HPD), with calendar years, most probable locations, and state posterior probabilities (spp) of the 77 complete genomes of West Nile virus.

NODE	CLADE	SUBCLADE	TMRCA^1^		YEARS		LOCATION	SPP^3^
			MEAN	CI^2^	MEAN	CI^2^		
**ROOT**	**EUROPE**		12.78	11–15.06	2002.22	1999.94–2004	HU	0.8
	**A**		8.6	7.3–10	2006.4	2005–2007.7	AT	0.52
		**A**_**IT**_	5.86	4.6–7.05	2009.14	2007.95–2010.4	IT	0.84
		**A**_**IT1**_	2.91	2.53–3.25	2012.08	2011.75–2012.47	IT	1
		**A**_**IT2**_	2.93	2.51–3.33	2012.07	2011.67–2012.49	IT	1
	**B**		7.55	6.15–9.04	2007.45	2005.96–2008.85	HU	0.66

^1^ tMRCA: Time of the most Recent Common Ancestor

^2^CI: Credibility interval

^3^SPP: State posterior probability

The analysis of migration rates showed significant linkages between Austria and the Czech Republic (2lnBF = 148.5), between Greece and Hungary (2lnBF = 5.78), and between Hungary and Serbia (2lnBF = 3.44).

**Fig 3:**
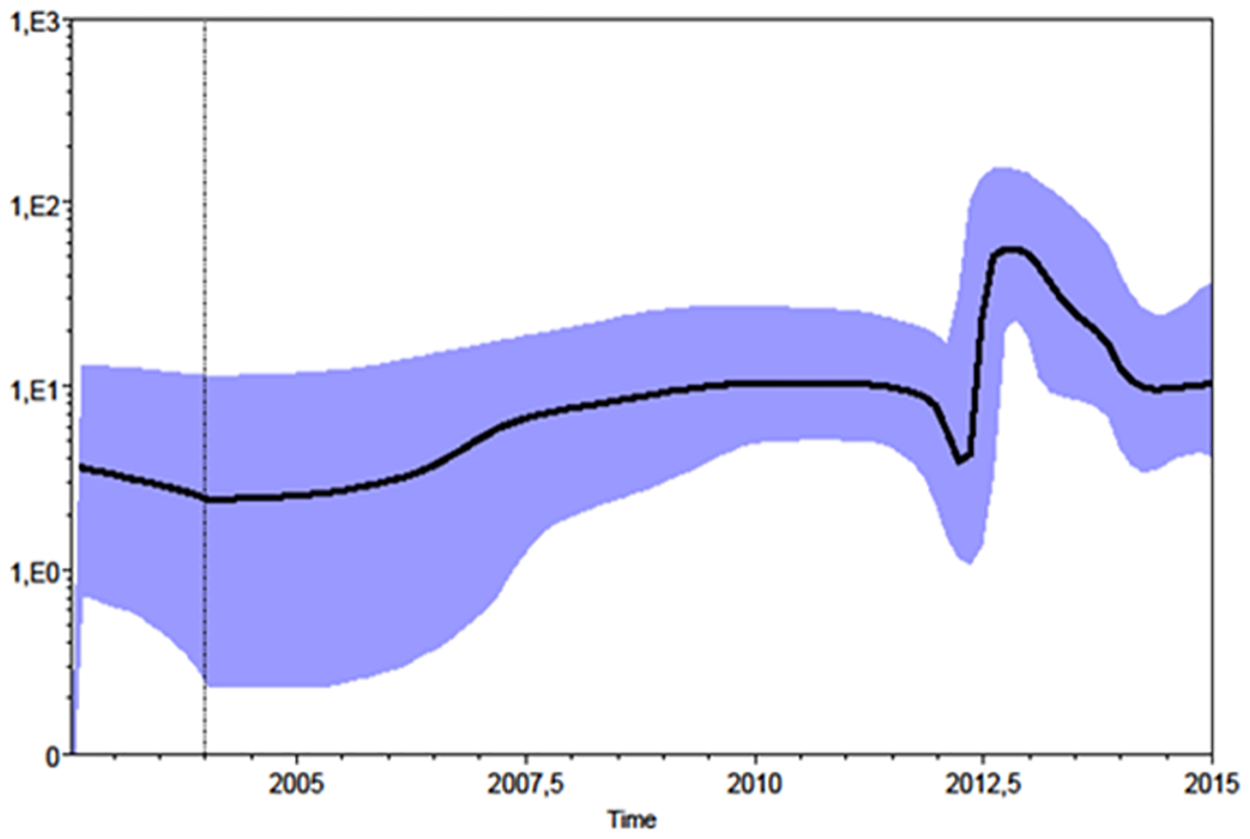
Population dynamics analysis of the European monophyletic clade of WNV-2. Bayesian skyline plot (BSP). The effective number of infections is indicated on the Y axis, and time on the X-axis. The coloured area corresponds to the credibility interval based on the 95% highest posterior density interval (HPD).

The Skyline plot (Fig 3) suggests that, after entering Europe in 2002, the epidemic remained relatively constant in size until 2012, when there was an exponential growth of 1 log followed by a partial decrease.

### Continuous phylogeography of the WNV-2 epidemic in Italy

The spread of WNV-2 in Italy was also investigated in continuous space by comparing the strict Brownian diffusion model with the relaxed random walk (RRW) models (Cauchy and Gamma distributions). The BF test showed that the Cauchy-distributed model outperformed all of the others (Cauchy distribution RRW *vs* homogenous BD: 2lnBF = 4 by PS and 5.78 by SS; Cauchy distribution RRW *vs* Gamma distribution RRW: 2lnBF = 16.06 by PS and 14.18 by SS; and Cauchy distribution RRW *vs* log normal RRW: 2lnBF = 10.04 by PS and 11.22 by SS).

The Italian isolates formed two highly significant sub-clades: one including the majority of the sequences obtained in the eastern part of the Po valley (mainly Rovigo and Ferrara) and the other the majority of the isolates obtained in the western part of the valley (mainly Lodi, Pavia and Cremona); the strains isolated in the central region of the Po valley (Mantua, Modena and Parma) were distributed in both sub-clades. One single isolate from Ancona was at the outgroup of the tree.

**Fig 4:**
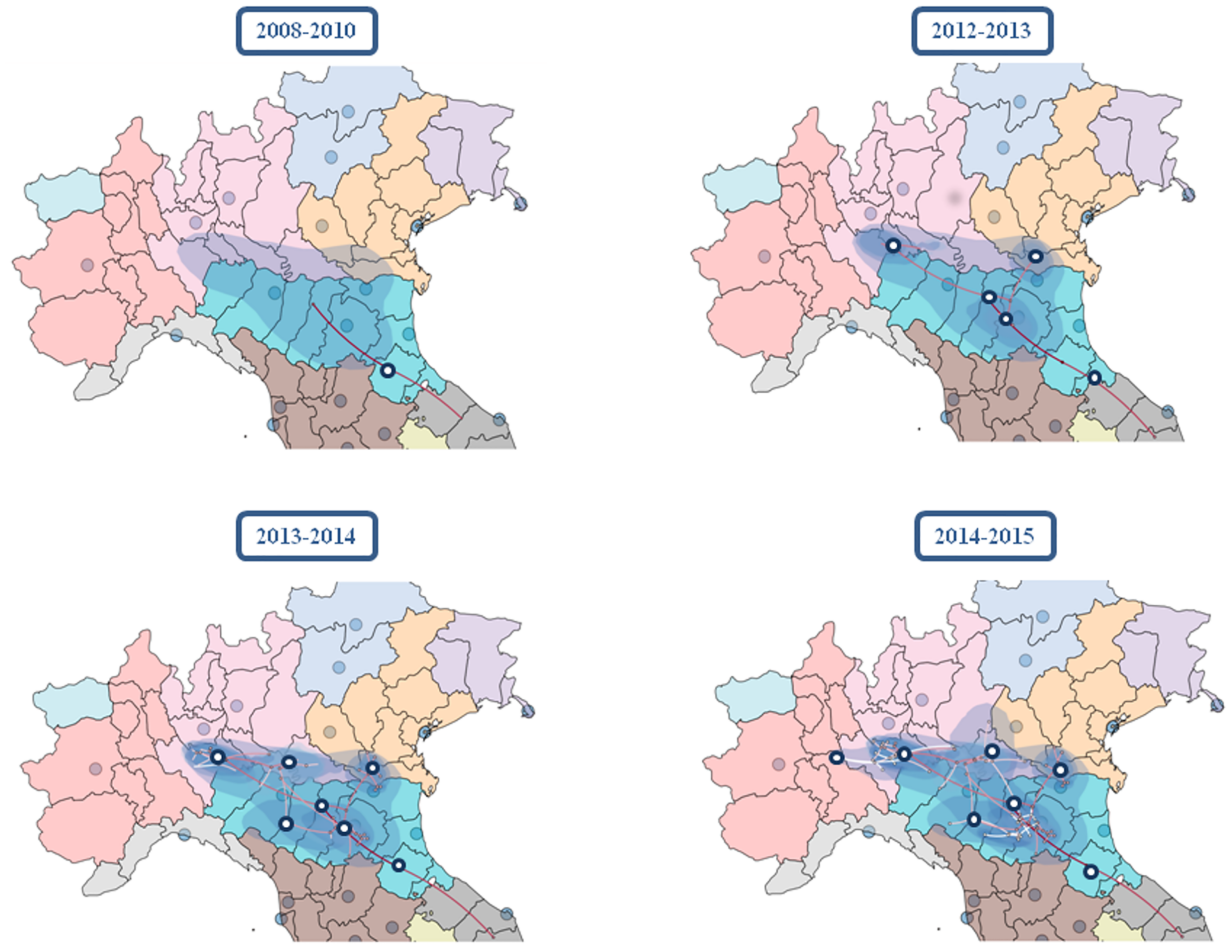
Spatio-temporal dynamics of the West Nile virus epidemic in Italy. The inferred spatiotemporal dynamics of WNV-2 in Northern Italy. The figure summarize the most significant migration links in the interested area. More detailed results are reported in S1 Video.

The coordinates of the tree root estimated by means of continuous phylogeography were *44°*, *04’N* and *11°*, *58’E*, falling halfway between Ancona (the place of the first Italian isolate) and the Po river. The tMRCA estimate was 7.7 YA (95% HPD = 4.81–10.9), which suggests that WNV-2 entered Italy in 2008, and reached an area between Parma, Reggio Emilia and Mantova in 2010. The epidemic then spread simultaneously westward and eastward (Fig 4). The eastern spread reached a region between Modena and Bologna in 2012 and was then redirected northward to reach Rovigo in the area of the Po delta; interestingly, this pathway apparently disappeared in 2013. The western path rapidly spread the virus to the area of Piacenza (in 2012) and Cremona, overflowed into the area of Lodi, Pavia and Milan in 2013, reached the border between Lombardy and Piedmont in 2014, and then moved southward to reach the region between Modena and Bologna once again in 2015.

On the whole, the virus spread throughout an area of about 244 km from east to west and 130 km from north to south on either side of the River Po over a period of about eight years (from 2008 to 2015). The estimated diffusion rate of the epidemic was about 42.69 km/year (95% HPD 27.60–57.69 km/year).

## Discussion

In the present study, we observed a high level of homogeneity of WNV-2 European strains, which segregated into a main monophyletic clade, with the exception of a small monophyletic group including isolates obtained in eastern Europe and in Italy [15].

In 2011, we published an article describing the phylogeography of WNV-1a in Europe, which highlighted the genetic heterogeneity of the virus particularly in eastern Europe [40]. These findings raised the hypothesis of various introductions of the virus along two main routes (eastern and western) probably carried by migrating birds such as white storks [40].

These data suggest that WNV-2 has very different eco/epidemiological characteristics, and suggests that this lineage came out of Africa more recently remaining more conserved than WNV-1a.

The temporal and spatial reconstruction of the eco/epidemiological history of the major European clade of WNV-2 on the basis of phylogeographical analysis were in line with the available epidemiological documentation. The tMRCA estimates suggest that this clade entered Europe as a result of a single or at most a few penetration events in 2002 (2000–2004), at least two years before it was first isolated in Hungary in 2004.

The migration rates estimated in our phylogeographical analysis suggested that, after entering Hungary, WNV-2 spread through central Europe westwards to Austria, and eastwards towards Greece. Both localities were reached around 2007 even if WNV-2 infections in humans or horses have only been documented in Austria since 2014, and the widespread outbreak in Greece occurred in 2010.

These data suggest the need for a period of time of enzootic circulation in Europe involving only reservoirs and vectors before the extension of the epidemic to dead-end hosts as it is confirmed by a sero-epidemiological study showing the presence of anti-flaviviral IgG in human samples collected in rural Northern Greece at least three years before the epidemic [41].

Analysis of the phylogeographical tree showed that the strains circulating in Greece formed a single highly significant clade (B), thus still suggesting a single penetration event, followed by local amplification every summer until at least 2014, when Greece reported for the last year WNV-2 infections. The westward migration was from Austria to the Czech Republic and Italy in 2010, and Serbia in 2012. Serbia was also involved by the Greek strain, thus suggesting its position as a crossroads in the spread of the virus. In brief, our phylogeographical analysis of the European migrations of WNV-2 indicates Hungary and Austria as radiation centres (sources) of European WNV2, whereas countries such as Greece or Italy mainly acted as receiving areas (sinks).

In Italy WNV-2 was isolated for the first time in 2011 from a feverish patient admitted to a hospital in Ancona on the Italian Adriatic coast [18]. At the same time, it was also isolated from one patient during a small outbreak in Sardinia involving six patients with WNNDs, four of whom died (the WNV lineage could be characterised in only two cases [20, 42]). After 2013, WNV-2 spread rapidly, and subsequently became the only strain isolated in humans and animals/vectors in Italy [17]. During the same period, the incidence of infection greatly increased and more neurological cases were documented than ever before: between 2008 and 2011, the median incidence of WNND was 0.4/100,000 inhabitants, but this increased threefold (1.2/100,000) between 2012 to 2015, with peaks in 2013 (1.66/100,000) and 2015 (1.34/1000,000) [17]. At the same time, the virus spread westward and southward [17].

In order to study the origin and spread of WNV-2 in Italy between 2011 and 2015 in greater detail, we analysed the Italian isolates included in the more general dataset by a continuous phylogeographical method, using the geographical coordinates identifying each isolate [43].

The results of this analysis indicated that, after its entry to Italy from the east Adriatic coast in an estimated period close to 2007, WNV-2 reached a central region of the Po Valley (between Parma and Modena) by 2010. Then it spread in two directions: the first towards the north-east and the Po delta, which was reached in 2013, and the second towards the western part of the Po valley through Lombardy until it finally reached Piedmont, the westernmost edge of its diffusion in 2015. Interestingly, the eastern strain seemed to disappear in 2013, being replaced from the western one in the south Eastern area of Modena and Bologna.

In brief, we identified the main westward axis of migration represented by the Po valley between Veneto and Piedmont, from where the virus radiated to the south and north following the main tributaries of the Po (the Panaro or Secchia southward, and Mincio northward).

Our findings show the importance of integrated human, animal and vector surveillance and suggests that phylogenetic studies can contribute to reconstructing the origin and geographical spread of global and local WNV-2 epidemics.

Our findings allow three main conclusions to be drawn:

1. The genomic homogeneity of the European WNV-2 strain suggests its relatively recent arrival from Africa;
2. The viral strain was introduced into central Europe as a result of a locally amplified single penetration event [22], which supports the view of [44] that the role of migratory and autochthonous birds should be investigated in more detail;
3. The epidemiological pattern suggests that the virus circulates among reservoirs and vectors for several years before causing animal and human outbreaks, and so surveillance systems should be maintained in high risk areas even in the absence of human cases, and efforts should be intensified to define areas in which the possibility of human infection would justify the implementation of measures of early diagnosis and optimised mosquito control.

## Supporting information

S1 Table Accession numbers and codes of all sequences included in the study. (DOCX)

S1 Fig Map of Northern Italy showing the distinct cities interested by WNV-2 epidemics in the years 2008–2015. (TIF)

S2 Fig MCC tree of the 97 West Nile virus genomes reconstructed using MrBayes. The numbers on the branches represent posterior probabilities (see Materials and methods for details). The main significant clades/subclades are highlighted. The scale axis below the tree shows the number of expected changes per site.
(TIF)

S3 Fig Likelihood mapping of the West Nile virus sequences. Each dot represents the likelihoods of the three possible unrooted trees for each quartet randomly selected from the data set: the dots near the corners or sides respectively represent tree-like (fully resolved phylogenies in which one tree is clearly better than the others) or network-like phylogenetic signals (three regions in which it is not possible to decide between two topologies). The central area of the map represents a star-like signal (the region in which the star tree is the optimal tree). The numbers indicate the percentage of dots in the centre of the triangle.
(TIF)

S1 Video Animated visualization of the continuous pattern of WNV-2 dispersion in Italy in the years 2008–2015. (MP4)
