# Supplementary material for: Reconstructing the recent West Nile virus lineage 2 epidemic in Europe and Italy using discrete and continuous phylogeography
Source: PLoS One. 2017 Jul 5;12(7):e0179679. doi: 10.1371/journal.pone.0179679 (PMC5497961; doi:10.1371/journal.pone.0179679)
Supplement: S1 Table — (DOCX) [file pone.0179679.s001.docx]

| **Name** | **Accession Number** |
| --- | --- |
| 1UG@37 | M12294 |
| 2UG@37 | NC_001563 |
| 3UG@37 | AY532665 |
| 4HU@04 | DQ116961 |
| 5SN@90 | DQ318019 |
| 6CF@82 | DQ318020 |
| 8ZA@89 | EF429197 |
| 9ZA@01 | EF429198 |
| 10ZA@00 | EF429199 |
| 11ZA@58 | EF429200 |
| 12ZA@89 | EU068667 |
| 13RU@07 | FJ425721 |
| 14ZA@58 | HM147822 |
| 15MG@88 | HM147823 |
| 16CD@58 | HM147824 |
| 17GR@10 | HQ537483 |
| 18iAN@11 | JN858070 |
| 19ZA@08 | JN393308 |
| 20SR@12 | KC407673 |
| 21GR@12 | KF179639 |
| 22AT@08 | KF179640 |
| 23iROV@13 | KF588365 |
| 24HU@10 | KC496015 |
| 25SR@10 | KC496016 |
| 26iROV@13 | KF647248 |
| 27iROV@13 | KF647249 |
| 28iROV@13 | KF647250 |
| 29iPD@13 | KF647251 |
| 30iROV@13 | KF647252 |
| 31CY@68 | GQ903680 |
| 32ZA@77 | KM052152 |
| 33UK@80 | JX041631 |
| 34iROV@13 | KF823805 |
| 35RO@13 | KJ934710 |
| 36CZ@13 | KM203860 |
| 37CZ@13 | KM203861 |
| 38CZ@13 | KM203862 |
| 39CZ@13 | KM203863 |
| 40AT@14 | KM659876 |
| 44iMN@13 | KF823806 |
| 45GR@13 | KJ577738 |
| 46GR@13 | KJ577739 |
| 47GR@13 | KJ883341 |
| 48GR@13 | KJ883342 |
| 49GR@13 | KJ883343 |
| 50GR@13 | KJ883344 |
| 51GR@13 | KJ883345 |
| 52GR@13 | KJ883346 |
| 53GR@13 | KJ883347 |
| 54GR@13 | KJ883348 |
| 55GR@13 | KJ883349 |
| 56GR@13 | KJ883350 |
| 57AT@14 | KP109691 |
| 58AT@14 | KP109692 |
| 59iUD@14 | KT207791 |
| 60iVR@14 | KT207792 |
| 61iVR@14 | KP789956 |
| 62iMN@13 | KP789960 |
| 63iPV@14 | KP789959 |
| 64iPV@14 | KP789958 |
| 65iCR@14 | KP789957 |
| 66iVR@14 | KP789955 |
| 67iCR@14 | KP789954 |
| 68iPV@14 | KP789953 |
