# Supplementary figures and images for: Reconstructing the recent West Nile virus lineage 2 epidemic in Europe and Italy using discrete and continuous phylogeography
Source: PLoS One. 2017 Jul 5;12(7):e0179679. doi: 10.1371/journal.pone.0179679 (PMC5497961; doi:10.1371/journal.pone.0179679)

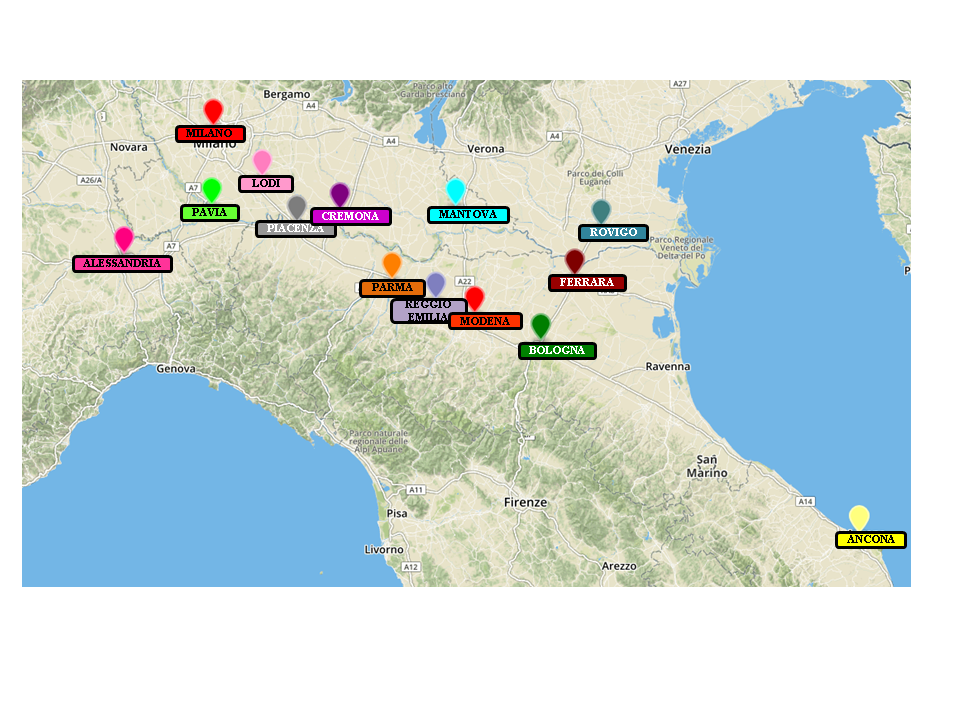

Supplement: S1 Fig — (TIF) [file pone.0179679.s002.tif]

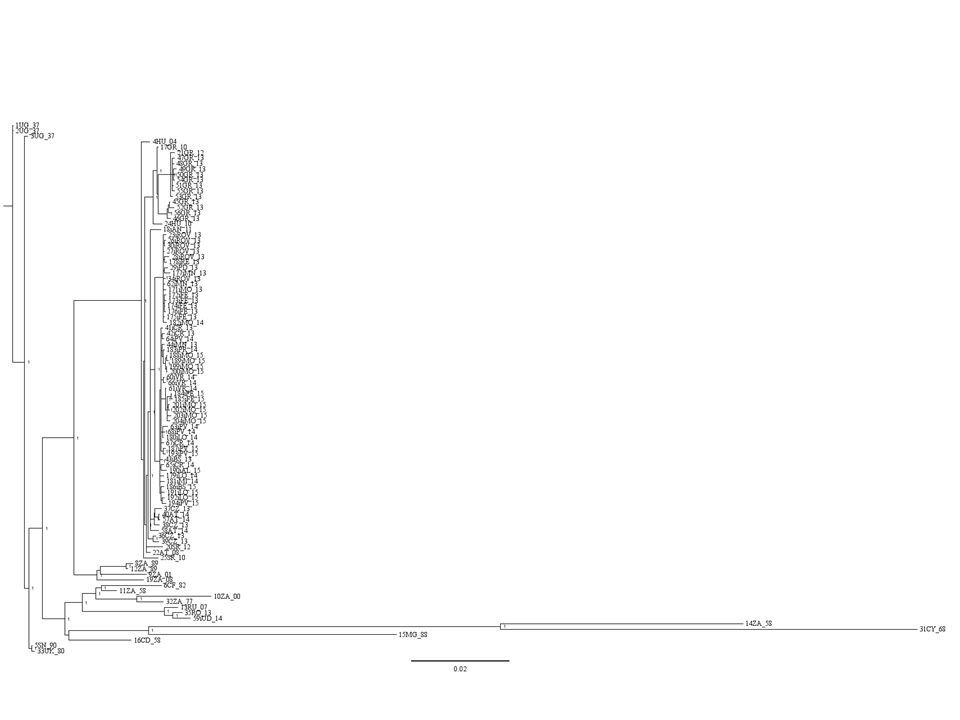

Supplement: S2 Fig — The numbers on the branches represent posterior probabilities (see Materials and methods for details). The main significant clades/subclades are highlighted. The scale axis below the tree shows the number of expected changes per site. (TIF) [file pone.0179679.s003.tif]

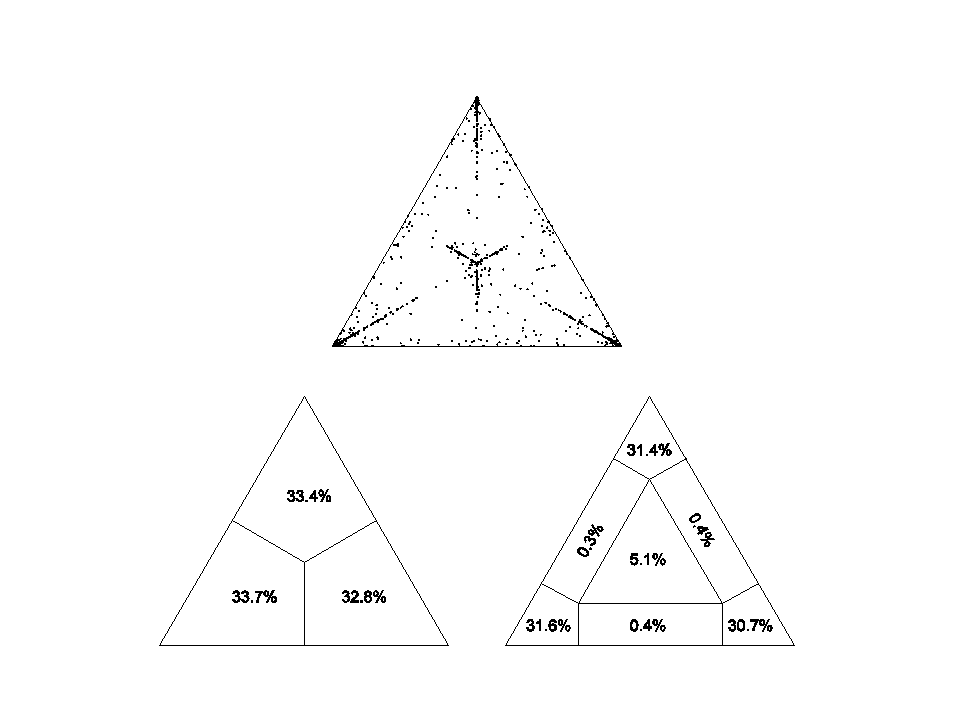

Supplement: S3 Fig — Each dot represents the likelihoods of the three possible unrooted trees for each quartet randomly selected from the data set: the dots near the corners or sides respectively represent tree-like (fully resolved phylogenies in which one tree is clearly better than the others) or network-like phylogenetic signals (three regions in which it is not possible to decide between two topologies). The central area of the map represents a star-like signal (the region in which the star tree is the optimal tree). The numbers indicate the percentage of dots in the centre of the triangle. (TIF) [file pone.0179679.s004.tif]
